# Unlocking the Therapeutic Potential of DNA-PKcs in Cancer: Comprehensive Insights into Mechanisms and Clinical Applications

**DOI:** 10.3390/cancers17172787

**Published:** 2025-08-26

**Authors:** Tong Zheng, Chao Sun, Cijun Yun, Hui Wang, Xiongxiong Liu

**Affiliations:** 1 Institute of Modern Physics, Chinese Academy of Sciences, Lanzhou 730000, China; zhengtong24@mails.ucas.ac.cn (T.Z.); sunchao@impcas.ac.cn (C.S.); yuncijun24@mails.ucas.ac.cn (C.Y.); 2 Gansu Provincial Isotope Laboratory, Lanzhou 730300, China; 3 University of Chinese Academy of Sciences, Beijing 100049, China; 4 Advanced Energy Science and Technology Guangdong Laboratory, Huizhou 516029, China

**Keywords:** DNA-PKcs, targeted therapy, DNA repair, tumor resistance

## Abstract

Cancer remains one of the most pressing global health challenges, with current therapies often hindered by limited efficacy and the emergence of resistance. The DNA-dependent protein kinase catalytic subunit (DNA-PKcs), a key regulator of DNA repair and cell cycle progression, plays a critical role in maintaining genomic stability, and growing evidence indicates its dysregulation in various cancers, with overexpression frequently associated with aggressive tumor phenotypes. To evaluate DNA-PKcs as a therapeutic target, we systematically analyzed literature from PubMed and Web of Science (2000–2024) using keywords including DNA-PKcs, targeted therapy, DNA repair, and tumor resistance following PRISMA guidelines, with 185 of 1250 initial records meeting inclusion criteria after screening. The review explores the multifaceted roles of DNA-PKcs in tumor biology and resistance mechanisms, evaluates the current landscape of DNA-PKcs inhibitors, including their clinical progress and combination strategies with radiotherapy and chemotherapy, and discusses key determinants of therapeutic efficacy, such as tumor type and mutation status. Additionally, it uniquely integrates emerging insights into the roles of DNA-PKcs in immunomodulation and metabolism, critically assesses next-generation inhibitors, and proposes strategies to address remaining challenges. Through this comprehensive analysis, we highlight the therapeutic potential of DNA-PKcs inhibition as a novel strategy to circumvent treatment resistance, providing innovative insights for optimizing cancer management, especially for aggressive tumor subtypes, thereby advancing drug discovery efforts and paving the way for more effective therapeutic interventions in clinical practice.

## Simple Summary

Cancer remains a major global health challenge. This review focuses on DNA-PKcs, a protein that enables cancer cells to repair DNA damage caused by radiotherapy and chemotherapy, promoting tumor survival. We synthesize recent evidence on how DNA-PKcs drives tumor progression and therapy resistance, highlighting its potential as a therapeutic target. Current advances in DNA-PKcs inhibitors are examined, including their promise in combination therapies and hurdles in clinical development. Uniquely, this review connects DNA-PKcs to emerging roles in immunomodulation and cancer metabolism, evaluates next-generation inhibitors, and proposes solutions to overcome translational challenges.

## 1. Introduction

Cancer continues to pose a formidable threat to global health, with the World Health Organization (WHO) reporting nearly 10 million cancer-related deaths in 2020 alone. While significant progress has been made in surgical, radiation, and systemic therapies, critical challenges persist, including intrinsic and acquired treatment resistance [1,2], therapy-associated toxicities [3,4,5], and interpatient heterogeneity [6,7]. These limitations highlight the urgent need for novel, targeted therapeutic strategies to enhance treatment efficacy and patient survival [8].

The DNA-dependent protein kinase catalytic subunit (DNA-PKcs), a core component of the DNA-PK complex, plays a pivotal role in repairing DNA double-strand breaks (DSBs)—a critical mechanism underlying cancer cell survival and genomic instability. Notably, cancer cells exhibit heightened DSB repair capacity, enabling uncontrolled proliferation and resistance to genotoxic therapies [9,10]. Beyond its canonical role in DSB repair, DNA-PKcs regulates diverse cellular processes, including cell cycle progression and apoptosis. Importantly, DNA-PKcs is frequently overexpressed or hyperactivated in multiple malignancies, such as nasopharyngeal carcinoma [11], breast cancer [12], and gastric cancer [13], with preclinical studies demonstrating that its inhibition effectively suppresses tumor growth. These findings position DNA-PKcs as a compelling therapeutic target in oncology.

This review aims to critically evaluate the therapeutic potential of DNA-PKcs inhibition in cancer treatment. We will (1) elucidate the biological functions of DNA-PKcs in tumorigenesis and therapy resistance, (2) assess the current landscape of DNA-PKcs inhibitors and their clinical progress, and (3) discuss the rationale for combining DNA-PKcs-targeted therapies with radiotherapy, chemotherapy, and emerging modalities. By synthesizing these insights, we seek to underscore DNA-PKcs as a high-value target and provide a roadmap for future research to optimize cancer therapeutics.

## 2. The Structure of DNA-PKcs

**Figure 1:**
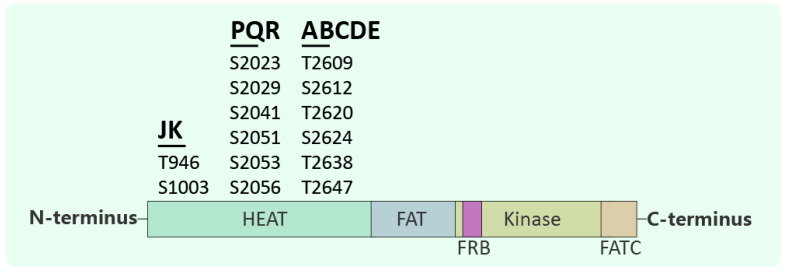
Domain architecture and functional phosphorylation sites of DNA-PKcs. Schematic representation of the major structural domains of DNA-PKcs, including the N-terminal HEAT repeats, FAT (FRAP-ATM-TRRAP), FRB (FKBP12-rapamycin binding), kinase, and C-terminal FATC domains. Key phosphorylation clusters (JK, PQR, and ABCDE) that regulate DNA-PKcs activity and function in DNA damage response. Phosphorylation at these sites modulates DNA-PKcs activation, autophosphorylation, and interaction with repair machinery.

DNA-PKcs, encoded by the PRKDC gene, is a 460 kDa multifunctional serine/threonine kinase belonging to the phosphatidylinositol 3-kinase-related kinase (PIKK) family, which includes other DNA damage response kinases like ATM and ATR [14,15]. This large protein (4128 amino acids) features an N-terminal HEAT repeat domain that forms an open ring structure essential for DNA-PK complex assembly and non-homologous end joining (NHEJ), with its binding affinity enhanced by a leucine-rich region [16,17,18]. The C-terminal region contains characteristic PIKK family domains, including a catalytic kinase domain that phosphorylates substrates involved in NHEJ, cellular metabolism, and apoptosis regulation [16,19,20]. Key regulatory domains include the FAT and FATC regions that maintain protein stability and facilitate substrate interactions, as well as the FRB domain which mediates mTOR binding for metabolic regulation [16,19,20] (Figure 1). Importantly, structural studies have identified conserved phosphorylation clusters (JK, ABCDE, and PQR) that critically regulate DNA-PKcs activation, repair complex assembly, and NHEJ efficiency [19,20,21,22], highlighting the sophisticated molecular architecture that enables its diverse cellular functions.

## 3. The Function of DNA-PKcs in Tumor Biology

### 3.1. The Classical Function of DNA-PKcs

DNA-PKcs plays pivotal roles in multiple DNA repair pathways, with its most well-characterized function in non-homologous end joining (NHEJ). Upon DNA double-strand break (DSB) formation, DNA-PKcs is recruited to damage sites where it complexes with Ku70/Ku80 to form DNA-PK [23,24,25]. This complex initiates NHEJ by recognizing broken DNA ends and facilitating their repair through autophosphorylation and substrate phosphorylation [26,27,28,29,30,31].

Beyond NHEJ, DNA-PKcs is essential for V(D)J recombination, the process that generates antibody diversity in developing lymphocytes [32,33]. It mediates DSB recognition and processing during immunoglobulin gene rearrangement while regulating key factors including Artemis and XRCC4 through its kinase activity [34,35,36].

**Figure 2:**
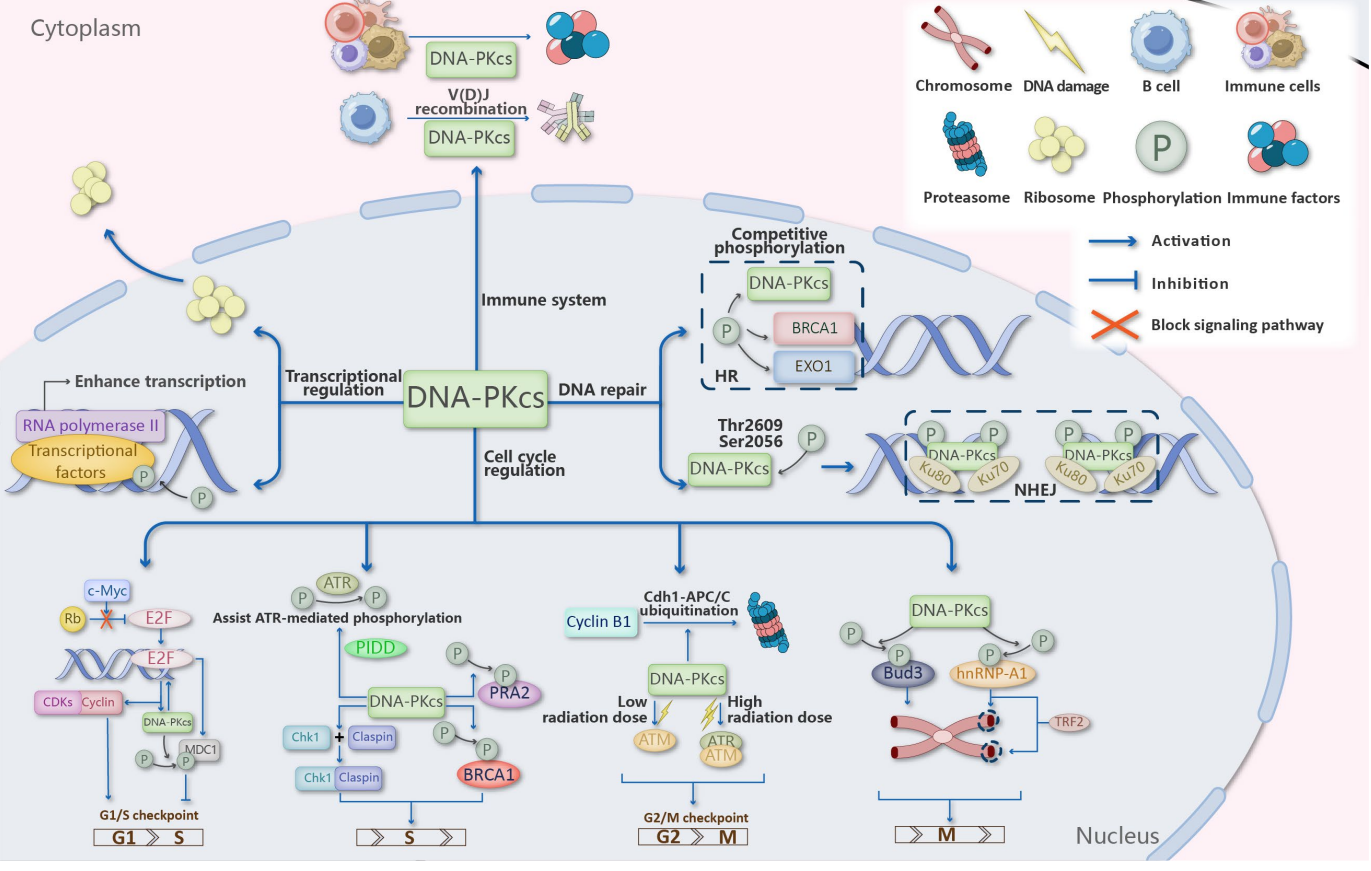
DNA-PKcs plays complex and indispensable roles extending far beyond its canonical functions, including DNA repair, cell cycle regulation, transcriptional control, and immune modulation. In DNA repair, it is recruited to double-strand breaks (DSBs) via the Ku70/Ku80 complex, where phosphorylation at Thr2609/Ser2056 clusters activates non-homologous end joining (NHEJ) while competitively inhibiting homologous recombination (HR) through BRCA1 and EXO1 phosphorylation. For cell cycle regulation, DNA-PKcs attenuates ATM-mediated G1/S arrest by phosphorylating MDC1, ensures proper chromosome segregation by modulating Cyclin B1 stability through the Cdh1-APC/C pathway and phosphorylating Bub3, and stabilizes replication forks via ATR and PIDD to prevent replication stress during S phase. In transcriptional regulation, it enhances RNA polymerase II activity and facilitates transcription factor recruitment. In immune modulation, it is essential for generating antigen receptor diversity and modulating immune factor expression, highlighting its multifaceted roles in cellular homeostasis.

DNA-PKcs also influences the choice between NHEJ and homologous recombination (HR) repair pathways. Through competitive phosphorylation with other PIKK family members, it modulates HR proteins such as BRCA1 and EXO1 [1,37,38,39,40]. While its role in single-strand break repair remains less understood, emerging evidence suggests single-stranded DNA can activate DNA-PKcs kinase function [41]. These DNA repair functions are particularly crucial for cancer cells, which depend on DNA-PKcs to maintain genomic stability during rapid proliferation and increased DNA damage burden (Figure 2).

### 3.2. Cell Cycle Regulation by DNA-PKcs

DNA-PKcs plays a critical role in coordinating cell cycle progression through multiple regulatory mechanisms (Figure 2). As a key DNA damage sensor, it modulates checkpoint responses at both G1/S and G2/M transitions. Through phosphorylation of MDC1, DNA-PKcs attenuates ATM-mediated G1/S checkpoint activation [42,43], while simultaneously regulating Cyclin B1 stability via the Cdh1-APC/C ubiquitination pathway to control G2/M transition [44]. Notably, DNA-PKcs overexpression can trigger Chk2 activation, leading to centrosome amplification and G2/M checkpoint disruption [45].

The kinase exerts broad influence over cell cycle progression through the c-Myc/E2F axis. By maintaining c-Myc stability [46], DNA-PKcs indirectly regulates cyclin D/CDK4/6 expression and subsequent pRb-E2F signaling [47,48,49]. Reciprocal regulation occurs through E2F binding sites in DNA-PKcs promoter regions [50], suggesting a feedback mechanism. During S phase, DNA-PKcs phosphorylates critical substrates including PRA2 and BRCA1 [38,51], while participating in Chk1-Claspin complex formation to maintain genomic stability [52]. Its functional interplay with other PIKK members is dose-dependent, with low radiation activating ATM and higher doses engaging both ATM and ATR for G2/M checkpoint control [53].

DNA-PKcs also safeguards chromosomal integrity throughout the cell cycle. It collaborates with PIDD to stabilize replication forks through ATR-mediated phosphorylation [54,55], while suppressing aberrant chromosome rearrangements via DNA end protection [56]. During mitosis, DNA-PKcs contributes to accurate chromosome segregation through Bub3 phosphorylation [57] and maintains telomere stability via interactions with TRF2 [58,59] and hnRNP-A1 phosphorylation [60].

While these findings establish DNA-PKcs as a master regulator of cell cycle progression, the complete mechanistic remains to be elucidated. Further investigation of these pathways may yield novel therapeutic strategies for cancer treatment by targeting DNA-PKcs-mediated cell cycle control.

### 3.3. DNA-PKcs in Tumor Immunogenicity

**Figure 3:**
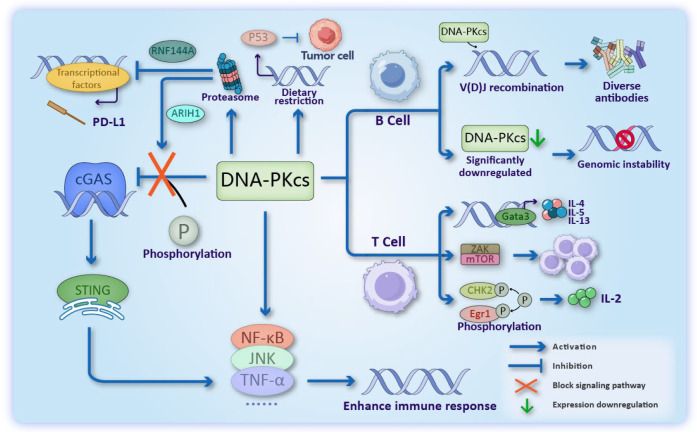
Role of DNA-PKcs in Tumor Immunogenicity. It modulates immune responses through: (1) Transcriptional regulation of immune-related factors (2) Phosphorylation-mediated inhibition of cGAS, thereby suppressing the cGAS/STING innate immune pathway (counteracted by ARIH1 through proteasomal degradation of DNA-PKcs) (3) RNF144A-dependent regulation of PD-L1 expression, potentially limiting immune evasion in cancer and (4) In B cells, DNA-PKcs is essential for immunoglobulin class switch recombination (CSR), and its deficiency leads to genomic instability. In T cells, DNA-PKcs modulates Gata3-dependent cytokine production, sustains proliferative capacity by activating the ZAK/mTOR signaling axis, phosphorylates CHK2 and Egr1, thereby enhancing IL-2 expression and promoting T cell activation.

The emerging role of DNA-PKcs in immunoregulation presents new opportunities for cancer immunotherapy (Figure 3). As a key mediator of V(D)J recombination, DNA-PKcs deficiency results in severe immunodeficiency, characterized by reduced immune cell populations and radiosensitivity in murine models [61,62,63,64]. Mechanistically, DNA-PKcs regulates T cell function through multiple pathways: (1) modulating Gata3-mediated cytokine production (IL-4, IL-5, IL-13) in CD4+ T cells [65]; (2) activating ZAK/mTOR signaling to maintain senescent T cell proliferation [66]; and (3) phosphorylating CHK2 and Egr1 (Early growth response protein 1) to upregulate IL-2 expression [67,68].

In B cell immunity, DNA-PKcs is indispensable for immunoglobulin class switch recombination (CSR). DNA-PKcs-deficient B cells exhibit defective CSR with increased alternative end joining (aEJ) activity, leading to genomic instability through non-productive translocations and deletions [69,70,71,72]. Phosphorylation at Thr2609 appears critical for maintaining canonical NHEJ during CSR [72], suggesting therapeutic potential for modulating DNA-PKcs activity.

The kinase also interfaces with innate immunity through the cGAS-STING pathway [73,74]. While DNA-PKcs phosphorylates and inhibits cGAS [75,76], its degradation by ARIH1 enhances STING-dependent immune activation [76]. This regulatory interplay extends to other immune pathways (NF-κB, JNK, TNF-α) [77,78,79,80], though context-dependent effects are observed—dietary restriction activates DNA-PKcs/p53-mediated tumor suppression [81,82,83], highlighting its pleiotropic functions.

Notably, DNA-PKcs interacts with immune checkpoint regulation [84,85]. It maintains PD-L1 expression [86,87,88], while the E3 ligase RNF144A coordinately degrades both DNA-PKcs and PD-L1 [87,89]. These findings position DNA-PKcs as a promising target for combination immunotherapy, though its complex immunomodulatory roles necessitate careful therapeutic development.

### 3.4. Transcriptional Regulation by DNA-PKcs

DNA-PKcs plays a pivotal role in transcriptional regulation through multiple mechanisms (Figure 2). Initial studies in vivo experiments show that DNA-PKcs is essential for p53-dependent high-level transcription [90]. This kinase regulates RNA polymerase II (RNAPII) activity by facilitating its phosphorylation and formation of transcription factor complexes [91,92], while also being recruited by hypoxia-inducible factor-1 to reactivate transcription under hypoxic conditions [93,94,95]. Additionally, DNA-PKcs participates in ribosomal biogenesis through phosphorylation of hnRNP and pre-rRNA [96,97], and modulates the activity of various transcription factors including: (1) Core transcriptional regulators (TBP, USF, FoxA2) [98,99,100]; (2) Metabolic/survival mediators (NF-κB, Myc, Sp1) [101,102,103]; (3) Immune response controllers (Egr1, NFAT) [68].

Dysregulation of these DNA-PKcs-mediated processes contributes to multiple pathologies such as tumorigenesis, renal, heart and neurological diseases [104,105,106]. AR variants (AR-Vs), which are unconventional nuclear hormone receptor family transcription factors, have their transcriptional activity enhanced by DNA-PKcs binding to the transcriptional elements and DNA-PKcs also participates in the mRNA processing of AR-Vs together with RNA-binding protein RBMX, eventually leading to increased expression of AR-Vs in advanced prostate cancer and resistance to traditional AR-targeted therapy [107].

### 3.5. Elevated DNA-PKcs Levels as a Driver of Tumor Progression

**Figure 4:**
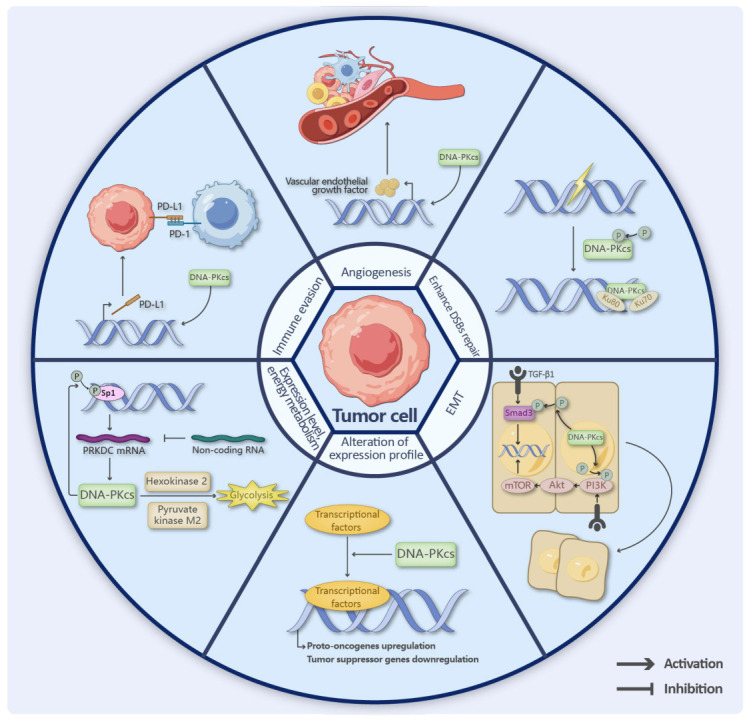
DNA-PKcs upregulation drives multifaceted oncogenic pathways in cancer progression. DNA-PKcs forms a complex with Ku70/Ku80 at double-strand breaks (DSBs), where it phosphorylates Thr2609/Ser2056 clusters to activate NHEJ repair. Through the TGF-β/Smad3 and PI3K/Akt/mTOR pathways, DNA-PKcs triggers epithelial–mesenchymal transition (EMT). It also phosphorylates Sp1, which promotes PRKDC mRNA expression. As non-coding RNA effects on PRKDC mRNA decrease, DNA-PKcs levels rise further, forming a positive feedback loop. This process activates glycolysis through enzymatic actions, supplying energy to cancer cells. Additionally, DNA-PKcs promotes vascular endothelial growth factor expression, supporting angiogenesis. High DNA-PKcs levels are also associated with PD-L1 overexpression, the combination of DNA-PKcs inhibitors with immunotherapy represents a promising therapeutic strategy.

Elevated DNA-PKcs expression is strongly correlated with enhanced metastatic potential and poor clinical prognosis across multiple cancer types, functioning through both direct and indirect oncogenic mechanisms (Figure 4). At the direct level, DNA-PKcs facilitates cancer cell survival by promoting efficient repair of DNA double-strand breaks (DSBs) through increased protein expression and/or enhanced kinase activity. Indirectly, it drives tumor progression by orchestrating metabolic reprogramming, modulating gene expression networks, and reshaping the tumor microenvironment [1,4,62].

The transcriptional and post-transcriptional regulation of DNA-PKcs plays a pivotal role in cancer progression, as exemplified by its interaction with transcription factor Sp1 in hepatocellular carcinoma, colorectal cancer, and gliomas. This interaction not only enhances PRKDC promoter binding and transcription but may also establish a self-reinforcing loop through Sp1 phosphorylation by DNA-PKcs, thereby promoting tumor aggressiveness and therapy resistance [12,102,108,109,110,111]. Similar regulatory mechanisms have been observed in cervical cancer [112]. Non-coding RNAs further contribute to this complex regulatory network, with miR-101 downregulation increasing PRKDC expression [113], while ScaRNA2 and linc00312 inhibit DNA repair by binding DNA-PKcs [114,115]. The lncRNA LINP1 facilitates DNA-PK complex formation during NHEJ [116], and circ_PRDKC modulates drug resistance and cell cycle checkpoints through miRNA sponging [117,118]. DNA-PKcs also forms critical interactions with other molecules, such as the DNA-PKcs/Survivin heterotetramer that enhances DSB repair while suppressing apoptosis [119,120], and its autophosphorylation at Thr2609 and Ser2056 regulates DSB repair efficiency and mitotic progression, though the full implications of these modifications remain incompletely understood [22,72,121]. The regulatory complexity is further compounded by phosphorylation events mediated by other kinases, particularly members of the PIKK family [122].

Recent advances have illuminated DNA-PKcs’ role in metabolic reprogramming and transcriptional control across various cancers. In castration-resistant prostate cancer (CRPC), it upregulates glycolysis through hexokinase 2 and pyruvate kinase M2 activation [123] while simultaneously promoting androgen receptor (AR) transcription [107]. Small cell lung cancer (SCLC) studies reveal its cooperation with OCT4 to activate Myc [101], and in pancreatic cancer, it modulates survival through the PI3K/Akt/mTOR pathway [46,110]. DNA-PKcs-mediated Smad3 phosphorylation enhances TGF-β1-induced epithelial–mesenchymal transition (EMT), contributing to metastasis in colorectal, gastric, and liver cancers [124,125,126,127]. Notably, in gliomas, the aberrantly expressed TRIM24 recruits DNA-PKcs and PHAX, leading to TRIM24 phosphorylation and subsequent oncogenic transcriptome alterations [128].

Although research on the role of DNA-PKcs in the tumor microenvironment (TME) remains limited, several significant findings have emerged that warrant attention. Studies demonstrate that DNA-PKcs facilitates Drp1 phosphorylation, a key mitochondrial fission protein, thereby accelerating mitochondrial fragmentation and modulating angiotensin II (Ang II)-induced vascular remodeling [129]. In TPEN-treated colon cancer cells, DNA-PKcs cooperates with Chk1 to mediate ROS-dependent DNA damage [130], revealing the therapeutic potential of heavy metal chelators in oncology. Notably, metastatic triple-negative breast cancer (TNBC) often develops immunotherapy resistance due to compromised immune cell infiltration and impaired inflammatory responses within the TME [131]. Intriguingly, DNA-PKcs inhibition has been shown to enhance immune cell infiltration and upregulate immunomodulatory factors, thereby reshaping the TME and potentiating anti-tumor immunity in TNBC [132]. Another mechanistic study in gastric cancer identified that hsa_circ_0136666 competitively binds miR-375-3p to elevate DNA-PKcs expression, consequently inducing PD-L1 overexpression and facilitating immune evasion88. Growing evidence further implicates DNA-PKcs in regulating oxidative stress responses, Akt signaling, and Smad/TGF-β1 pathways, which collectively influence tumor invasion and metastatic potential [91,92,124,127,133,134].

Emerging clinical observations suggest an unexpected association between DNA-PKcs downregulation and gastric cancer progression. A comprehensive clinicopathological analysis revealed that gastric cancer patients lacking DNA-PKcs expression exhibited significantly worse survival outcomes compared to those with preserved expression (*p* = 0.004) [135]. Moreover, DNA-PKcs loss showed strong correlation with advanced disease stage (*p* < 0.001) [135], implying potential tumor-suppressive functions that merit further investigation. While most therapeutic strategies targeting DNA-PKcs overexpression have shown encouraging clinical efficacy [136,137,138,139,140], the protein’s pleiotropic functions underscore the need for more sophisticated research approaches. Future investigations employing single-cell omics and spatial omics technologies will be crucial for elucidating the complex biology of DNA-PKcs and optimizing targeted therapeutic interventions.

## 4. Therapeutic Strategies Targeting DNA-PKcs

Given the critical role of DNA-PKcs in cancer cell proliferation and treatment resistance, considerable efforts have been devoted to developing DNA-PKcs inhibitors, many of which are now under preclinical and clinical evaluation. Targeting DNA-PKcs offers a promising strategy to overcome therapeutic resistance—a major challenge in oncology that frequently leads to tumor relapse and disease progression.

Early-generation inhibitors such as NU7026 and NU7441 attracted substantial interest due to their high selectivity for DNA-PKcs inhibition, yet they remain confined to preclinical studies [141,142]. In recent years, however, a new wave of next-generation DNA-PKcs inhibitors has entered clinical trials, either as standalone therapies or in combination with radiotherapy and chemotherapy. The following section highlights recent advances in DNA-PKcs-targeted therapies, focusing on their synergistic effects with conventional treatments and key factors influencing therapeutic efficacy (Table 1).

### 4.1. Current Landscape of DNA-PKcs Inhibitors

Preclinical studies have identified NU7026 and NU7441 as promising DNA-PKcs inhibitors, yet their clinical translation has been hindered by pharmacokinetic limitations including rapid blood clearance, poor oral bioavailability, and off-target effects on the mTOR/PI3K pathway [143,144]. These challenges have spurred the development of novel, more clinically viable inhibitors.

#### 4.1.1. Peposertib

Peposertib is a highly potent and selective small-molecule inhibitor of DNA-PKcs, currently under advanced clinical evaluation. Preclinical studies in cervical cancer xenograft models demonstrated significant target inhibition following oral administration. In the first-in-human Phase I trial (NCT02316197), 400 mg twice daily (BID) was established as the recommended Phase II dose (RP2D), with dose-limiting toxicities—primarily manageable gastrointestinal events—observed at 300 mg BID. Pharmacodynamic analyses confirmed target engagement, as evidenced by reduced phosphorylated DNA-PK levels in peripheral blood mononuclear cells, supporting its progression into combination therapy trials [145].

#### 4.1.2. CC-115

CC-115 is a promising dual-target inhibitor that selectively targets mTOR and DNA-PKcs, modulating the cell cycle and DSB repair, and it has shown efficacy in renal cancer and NSCLC cell lines [146,147,148]. In the first-in-human Phase I study, CC-115 showed notable efficacy and tolerability in patients with advanced tumors. The half-life and blood concentration were within acceptable ranges, and the RP2D was set at 10 mg BID. Remarkably, a patient with endometrial cancer achieved complete regression of lung and lymph node lesions after 10 cycles, which was maintained for four years. Additionally, the study suggests that CC-115 may be effective against a broader range of solid tumors than previously thought [149]. The Ib phase multi-center trial demonstrated the feasibility of combining 5 mg BID CC-115 with the AR inhibitor enzalutamide in patients with mCRPC. Notably, differences in PSA50 (Prostate-specific Antigen 50% decline from baseline, and the same applies to PSA90) and PSA90 between patients with and without PI3K pathway alterations underscored the importance of the PI3K pathway in this combination therapy [150].

#### 4.1.3. XRD-0394

XRD-0394 is an orally bioavailable dual inhibitor with high potency and specificity against ATM and DNA-PKcs. By disrupting these kinases in cancer cells, XRD-0394 enhances tumor sensitivity to radiotherapy (RT) [151].

#### 4.1.4. AZD7648

AZD7648 has shown promising potential in combination with RT, chemotherapy, and immunotherapy [152,153,154,155]. A completed Phase I/II trial (NCT03907969) evaluated AZD7648 in combination with pegylated liposomal doxorubicin (PLD), while another ongoing trial (NCT05116254) is investigating its synergy with RT.

#### 4.1.5. M9831 (VX-984)

M9831 enhances the radiosensitivity of cancer cells in a concentration-dependent manner, though its efficacy as a monotherapy requires further investigation [137,156]. A Phase I trial (NCT02644278) assessing M9831 in combination with PLD for advanced solid tumors has been completed.

#### 4.1.6. AsiDNA

Unlike conventional DNA-PKcs inhibitors, AsiDNA operates via a unique mechanism: it is a double-stranded DNA decoy that mimics DSBs, sequestering key NHEJ repair proteins, including DNA-PKcs. By depleting available DNA-PKcs, AsiDNA impairs the repair of endogenous DNA damage in cancer cells, ultimately triggering cell death [157,158,159]. This mechanism confers AsiDNA with a distinct advantage in overcoming drug resistance, making it a particularly promising therapeutic agent [157,160].

### 4.2. Combination Therapies Targeting DNA-PKcs (Table 2)

#### 4.2.1. DNA-PKcs Inhibitors and Radiotherapy

DNA-PKcs inhibitors enhance the efficacy of RT by disrupting DSB repair mechanisms, thereby increasing tumor radiosensitivity. This synergistic approach has demonstrated promising therapeutic potential across multiple preclinical models, including cervical cancer, oral squamous cell carcinoma, non-small cell lung cancer (NSCLC), and gliomas [155,161,162,163]. Beyond impairing DNA repair, DNA-PKcs inhibitors have been shown to modulate the TME under ionizing radiation, suppressing VEGF and HIF-1α expression in glioma cells and impeding microvascular endothelial cell proliferation and metastasis—a finding that opens new avenues for TME-targeted therapies [163].

Peposertib, for instance, exhibits enhanced tumor-targeting specificity when combined with RT, as evidenced in a melanoma brain metastasis model where alterations in DNA-PKcs phosphorylation and subcellular localization were observed [161,164]. Similarly, the dual ATM/DNA-PKcs inhibitor XRD-0394 improves RT response when co-administered with PARP inhibitors or topoisomerase I inhibitors [151]. An ongoing clinical trial (NCT05002140) is further evaluating this combinatorial strategy.

Additionally, the interplay between DNA-PKcs and RT has spurred interest in combining DNA-PKcs inhibitors with radionuclide therapy, with several clinical trials (NCT04071236, NCT04750954) currently investigating this approach [165,166]. These developments highlight the expanding therapeutic potential of DNA-PKcs inhibition in optimizing radiation-based cancer treatments.

**Table 2:** Combination Therapies Targeting DNA-PKcs.

Combination Type	Mechanism	Drugs/Therapies Involved	Research Progress	Key Findings
Radiotherapy	Disrupt DNA repair mechanisms to enhance radiosensitivity	Radiotherapy	Preclinical animal studies	Enhances Radiotherapy efficacy; expands radiation-based therapy potential; opens TME-targeted avenues.
	Regulate the expression of VEGF and HIF-1α to prevent cancer cell metastasis	Radiotherapy	Preclinical animal studies	
	PARP inhibitors or topoisomerase I inhibitors enhance radiosensitivity	PARP inhibitors or topoisomerase I inhibitors, Radiotherapy	NCT05002140, Phase I	
	Disrupt DNA repair mechanisms to enhance radiosensitivity	Radionuclide therapy	NCT04071236, Phase I, Phase II; NCT04750954, Phase I	
Chemotherapy	Induce DNA replication stress and impair damage repair pathways	Temozolomide	Preclinical animal studies	Multi-targeted; synergistic anti-tumor effects; potential to improve outcomes.
	Competitive receptor binding blocks oncogenic signaling	Neratinib		
	Inhibit androgen receptor signaling in prostate cancer	Enzalutamide		
	Weaken DNA repair ability to induce synthetic lethality	PARP inhibitors (Niraparib/Olaparib)		
Immunotherapy	DNA-PKcs overcomes PD-1/PD-L1 resistance by reprogramming TME and regulates PD-L1 expression.	Radionuclide therapy, Avelumab	NCT04071236, Phase I, Phase II	Strong mechanistic basis; synergizes with checkpoint blockade; overcome resistance potentially; potential immunotoxicity.
		Radiotherapy, Avelumab	NCT04068194, Phase I, Phase II	
Novel DNA-PKcs Inhibitor	Activating DNA-PK/p53/p21 pathway protects normal cells and enhances cancer cell death. Combined with PARP inhibitors, it induces synthetic lethality in cancer cells.	Olaparib	NCT05700669, Phase I, Phase II	Spares normal tissues, promotes their repair; low resistance propensity; synergizes with PARP inhibitors.
		Monotherapy	NCT03579628, Phase I	

#### 4.2.2. DNA-PKcs Inhibitors and Chemotherapy

The therapeutic potential of combining DNA-PKcs inhibitors with conventional chemotherapeutic agents has become an important focus in oncology research. This combination approach exerts synergistic anti-tumor effects through multiple mechanisms of action: (1) Temozolomide induces DNA replication stress and impairs damage repair pathways; (2) Neratinib blocks HER2-mediated oncogenic signaling through competitive receptor binding; (3) Enzalutamide suppresses androgen receptor signaling in prostate cancer; and (4) PARP inhibitors (Niraparib/Olaparib) induce synthetic lethality by compromising DNA repair capacity [111,167,168,169]. These mechanistic insights have driven substantial interest in developing combination regimens incorporating next-generation DNA-PKcs inhibitors like peposertib and AZD7648. Current research efforts are particularly focused on evaluating these agents in combination with both chemotherapy and radiotherapy, with promising preclinical data supporting their potential to overcome treatment resistance and improve therapeutic outcomes [148,152,155,170]. The multi-targeted nature of these combination strategies offers a compelling approach for addressing the complexity of cancer pathogenesis.

#### 4.2.3. DNA-PKcs Inhibitors and Immunotherapy

The multifaceted role of DNA-PKcs in immune regulation, particularly through its involvement in the cGAS-STING signaling pathway, interleukin-mediated responses, PD-L1 expression modulation, and immune cell function regulation, establishes a strong mechanistic foundation for combining DNA-PKcs inhibitors with immunotherapy. The most promising clinical potential appears to lie in combining these inhibitors with PD-1/PD-L1 checkpoint blockade, as evidenced by compelling preclinical data demonstrating synergistic antitumor effects [171]. Recent breakthroughs have elucidated DNA-PKcs’s critical function in macrophage biology, where it orchestrates DNA sensing mechanisms with mTORC2/Rictor/Myc-dependent proliferative signaling, revealing a novel therapeutic avenue for overcoming PD-1/PD-L1 resistance through tumor microenvironment reprogramming [172,173]. Preclinical evaluation of AZD7648 in combination with radiotherapy has demonstrated robust activation of type I interferon responses and significant enhancement of CD8+ T cell-mediated antitumor immunity [154], though careful consideration must be given to potential immunotoxicity profiles [174]. The concurrent modulation of the tumor microenvironment by both DNA-PKcs inhibitors and immunotherapy presents a unique opportunity to potentiate radiotherapy efficacy, prompting the initiation of several clinical trials (NCT04068194, NCT04071236) investigating this triple-combination therapeutic strategy that simultaneously targets DNA damage response pathways and immune checkpoint mechanisms.

#### 4.2.4. Novel DNA-PKcs Inhibitors

Emerging DNA-PKcs inhibitors demonstrate partial selectivity yet still exhibit considerable toxicity to healthy cells, compounded by pharmacokinetic challenges including poor metabolic stability and rapid clearance that have substantially hindered clinical development [164]. Intriguingly, research on AsiDNA has revealed a potentially superior therapeutic strategy—this novel agent uniquely induces G1/S cell cycle arrest in normal epithelial cells and fibroblasts through activation of the DNA-PK/p53/p21 pathway, a protective mechanism absent in p53-deficient tumor cells [157,175,176]. This differential effect not only spares normal tissues during ionizing radiation but actively promotes their DNA repair, making AsiDNA an exceptionally promising candidate for combination radiotherapy. Furthermore, AsiDNA synergizes remarkably with PARP inhibitors, inducing synthetic lethality in tumor cells while demonstrating significantly lower propensity for resistance development compared to conventional therapies [157,160,175,176]. Preclinical studies combining AsiDNA with olaparib have yielded particularly compelling results, showing both reduced toxicity in normal murine cells and enhanced tumor cell death through dual impairment of DNA repair pathways that leads to catastrophic accumulation of unrepaired DNA damage [157,177,178]. These findings have prompted ongoing clinical evaluation (NCT05700669) of this combination in recurrent solid tumors, while a separate trial (NCT03579628) is investigating AsiDNA monotherapy at 600mg in advanced solid tumors, with planned exploration of chemotherapeutic combinations to further potentiate its anticancer efficacy [179]. This next generation of DNA damage response inhibitors represents a paradigm shift in targeted cancer therapy by selectively exploiting tumor-specific vulnerabilities in DNA repair mechanisms.

### 4.3. Factors Influencing the Efficacy of DNA-PKcs Targeted Therapies

The therapeutic efficacy of DNA-PKcs-targeted interventions is governed by multiple interrelated biological and pharmacological factors. Foremost among these is the substantial variation in PRKDC gene mutation frequencies across cancer types, with colon (9.66%), gastric (9.63%), and endometrial cancers (9.27%) exhibiting markedly higher mutation rates compared to thyroid cancer (0.99%), glioblastoma (1.37%), and hepatocellular carcinoma (1.61%) [180]. Given that PRKDC encodes DNA-PKcs, these mutations can impair V(D)J recombination or kinase activity, with paradoxical effects on therapy response. For instance, kinase-activating mutations (e.g., Thr2609/Ser2056 clusters) may sensitize tumors to DNA-PKcs inhibitors [22,72], whereas truncating mutations could promote resistance. Structural studies further reveal that mutations in the FAT/FATC domains destabilize DNA-PKcs and modulate radiation sensitivity [181], highlighting the need for genomic profiling to guide inhibitor use.

Functional redundancy within the PIKK family further complicates treatment outcomes, as ATM/ATR can partially compensate for DNA-PKcs inhibition through phosphorylation-mediated regulation of downstream effectors [53,182]. Therefore, inhibiting DNA-PKcs, ATM, and ATR together can block the repair pathway and prevent tumor cells from developing drug resistance through compensatory mechanisms [183,184,185]. However, combining PIKK family inhibitors still raises significant safety concerns. The efficacy of ATM/DNA-PKcs co-inhibition is context-dependent, with variable sensitization observed across cell types [185]. Notably, therapeutic response correlates strongly with basal DNA-PKcs activity—low expression may diminish efficacy and promote resistance, independent of inhibitor dosage [182,183].

Emerging evidence suggests that mutations in TP53, ATM, or HRR genes (e.g., BRCA1/2) may predict sensitivity to DNA-PKcs inhibition. For instance, TP53-deficient tumors, reliant on NHEJ due to G1/S checkpoint loss, show heightened vulnerability to DNA-PKcs inhibitors like AZD7648 [154]. Similarly, ATM mutations confer synthetic lethality with DNA-PKcs blockade, as seen in preclinical models of XRD-0394 [151]. HR-deficient cancers (e.g., BRCA-mutated) exhibit synergistic lethality when DNA-PKcs inhibitors are combined with PARP inhibitors [157,177]. Clinical validation of these biomarkers is ongoing in trials such as NCT04071236 (DNA-PKcs + radionuclide therapy) and NCT05002140 (ATM/DNA-PKcs inhibition).

In conclusion, the therapeutic potential of DNA-PKcs inhibition is shaped by complex molecular determinants, including tumor-specific PRKDC mutation profiles, compensatory signaling within the PIKK family, and synergistic interactions with DDR deficiencies. While combination strategies show promise in overcoming resistance, their clinical translation requires careful consideration of biomarker-guided patient selection and toxicity management.

## 5. Conclusions

Recent research has unveiled DNA-PKcs as a multifunctional regulator in cancer biology, extending well beyond its classical role in NHEJ-mediated DNA repair. The protein’s diverse post-translational modifications enable its involvement in critical processes such as cell cycle regulation, cytotoxic stress responses, immune modulation, and oncogenic progression. Importantly, accumulating evidence strongly correlates heightened DNA-PKcs activity with cancer initiation, progression, and clinical outcomes, solidifying its therapeutic relevance as a molecular target. Although multiple DNA-PKcs inhibitors have entered clinical trials and shown encouraging results-both as monotherapies and in combination regimens—their development has faced substantial challenges.

The current lack of FDA-approved inhibitors stems from suboptimal pharmacokinetics, poor aqueous solubility, and dose-limiting toxicities that have prematurely halted several clinical programs. Moreover, tumor heterogeneity poses a major hurdle for achieving consistent therapeutic responses. The immunosuppressive tumor microenvironment can compromise DNA-PKcs-targeted therapies; for instance, PD-L1 upregulation and impaired immune cell infiltration in triple-negative breast cancer models have been shown to diminish treatment efficacy, though combining DNA-PKcs inhibition with immunotherapy may overcome this limitation. Overcoming these hurdles requires a multipronged approach: deeper mechanistic studies of DNA-PKcs regulation using systems biology methods, longitudinal analyses to identify optimal treatment windows, and rational combination strategies (e.g., with radiotherapy or immunotherapy) to enhance tumor specificity and overcome microenvironmental resistance. Crucially, current inhibitor designs show suboptimal engagement of the catalytic pocket, underscoring the need for structure-based drug optimization. Despite these challenges, the accumulated knowledge provides a strong foundation for developing next-generation inhibitors with improved selectivity and safety profiles, moving DNA-PKcs-targeted therapy closer to clinical application.

## Figures and Tables

**Table 1:** Clinical Trials Focusing on DNA-PKcs.

DNA-PKcs Inhibitos	Target	Combination Therapy	Clinical Trial Phase	Types of Cancer Treatment	Clinical Trial Registration
Peposertib	DNA-PKcs	Monotherapy	I	Advanced Solid Tumors	NCT02516813
		Radiotherapy	I	Glioblastoma, Gliosarcoma	NCT04555577
		Monotherapy	I/II	Locally Advanced Rectal Cancer	NCT03770689
		Radiotherapy, Avelumab	I/II	Cholangiocarcinoma, Gallbladder Carcinoma, Stage III Gallbladder Cancer AJCC v8, and 5 more	NCT04068194
		Radium-223 Dichloride, Avelumab	I/II	Metastatic Castration-Resistant Prostate Carcinoma, Metastatic Malignant Neoplasm in the Bone, Metastatic Malignant Neoplasm in the Lymph Nodes, and 1 more	NCT04071236
		Lutetium 177 Dotatate	I	Neuroendocrine Neoplasm	NCT04750954
CC-115	DNA-PKcs & mTOR	Monotherapy	I	Glioblastoma Multiforme, Glioblastoma Multiforme, Prostate Cancer, and 3 more	NCT01353625
		Temozolomide, Neratinib	II	Glioblastoma	NCT02977780
		Enzalutamide	I	Prostate Cancer, Castration Resistant Prostate Cancer	NCT02833883
XRD-0394	DNA-PKcs & ATM	Radiation: Palliative radiotherapy	I	Metastasis, Locally Advanced Solid Tumor, Recurrent Cancer	NCT05002140
AZD7648	DNA-PKcs	Radiotherapy	I	Soft Tissue Sarcoma Adult	NCT05116254
		Pegylated Liposomal Doxorubicin	I/II	Advanced Malignancies	NCT03907969
M9831	DNA-PKcs	Pegylated Liposomal Doxorubicin	I	Advanced Solid Tumor	NCT02644278
AsiDNA	DNA-PKcs	Monotherapy	I	Advanced Cancer	NCT03579628
		Niraparib, Olaparib	I/II	Ovarian Cancer	NCT04826198
		Monotherapy	I/II	Recurrent High-grade Glioma	NCT05394558
		Olaparib	I/II	Metastatic Castration-resistant Prostate Cancer, Recurrent Epithelial Ovarian Cancer, Breast Cancer	NCT05700669
